# Deep learning–based estimation of sagittal spinal alignment from coronal radiographs

**DOI:** 10.3389/fbioe.2026.1843705

**Published:** 2026-07-20

**Authors:** Tito Bassani, Riccardo Cecchinato, Marco Brayda-Bruno, Luca Maria Sconfienza

**Affiliations:** 1 Laboratory of Biological Structures Mechanics, IRCCS Istituto Ortopedico Galeazzi, Milan, Italy; 2 IRCCS Istituto Ortopedico Galeazzi, Milan, Italy; 3 Department of Biomedical Sciences for Health, University of Milan, Milan, Italy; 4 Unit of Diagnostic and Interventional Radiology, IRCCS Istituto Ortopedico Galeazzi, Milan, Italy

**Keywords:** artificial intelligence, deep learning, radiography, sagittal alignment, spine

## Abstract

**Introduction:**

Patient-specific sagittal alignment parameters are essential for accurate biomechanical modeling of the spine. These parameters are typically derived from biplanar radiographs, which, despite low-dose advancements, still involve cumulative radiation exposure. Deep learning has shown promise in automating radiographic analysis; however, prior approaches for cross-plane prediction—such as generating sagittal images from coronal views using generative adversarial networks (GANs)—have not achieved clinically acceptable accuracy. This study investigates an alternative strategy based on multiple-output regression (MOR) to directly predict sagittal parameters from coronal radiographs and evaluates its performance against GAN-based approach.

**Methods:**

A dataset of 449 adult subjects with EOS biplanar radiographs was used to train (313 subjects, 70% of dataset), validate and test (68 subjects, 15%, each) a MOR model based on convolutional neural networks. Six sagittal parameters (TK, LL, SS, PI, PT, SVA) were predicted from preprocessed coronal images. Model performance was assessed using absolute error metrics, regression analysis, Bland–Altman plots, and Lin’s concordance correlation coefficient (CCC). External inference was evaluated on an independent dataset of 69 adolescents with idiopathic scoliosis (AIS), enabling comparison between MOR predictions and parameters derived from GAN-generated sagittal images.

**Results:**

In the adult test set, median absolute errors ranged from 4° to 8° for angular parameters and 1.6 cm for SVA, with maximum errors up to 40° and 8.8 cm. CCC values varied from 0.20 (PI) to 0.69 (PT), indicating moderate agreement for some parameters and weak agreement for others. Regression and Bland–Altman analyses revealed both proportional and fixed biases. In the AIS dataset, MOR and GAN-based approaches showed comparable performance (median errors ∼7°), although GAN performed better for SVA (1 cm vs. 2 cm; p-value <0.001). Both methods exhibited reduced accuracy and lower CCC values compared with the adult dataset.

**Discussion:**

Direct prediction of sagittal alignment parameters from coronal radiographs using MOR yields limited accuracy and is affected by systematic biases. Comparable performance with GAN-based methods suggests that the primary limitation lies in the insufficient sagittal information contained in coronal projections. Current deep learning approaches are therefore inadequate to replace biplanar imaging for reliable sagittal assessment.

## 1 Introduction

The use of patient-specific alignment parameters is essential for accurately reproducing individual biomechanical spinal alignment in multibody musculoskeletal models and finite element parametric models, both under physiological conditions and in the presence of spinal deformities (Barba et al., 2021; Bassani et al., 2019; Bassani et al., 2024; Rauber et al., 2024; Carpenedo et al., 2025). Vertebral orientations in the anatomical planes, as well as alignment parameters within specific planes, are typically derived from the reconstruction or measurement of radiographic images acquired in the coronal and sagittal planes (Bassani et al., 2021; Barba et al., 2021; Bassani et al., 2017). The reliance on biplanar radiographs is well established, as they provide clinically accessible information on spinal geometry while minimizing the invasiveness and cost associated with advanced imaging modalities (Tomaiuolo et al., 2024).

In this context, the development of imaging systems such as slot-scan radiography and EOS low-dose biplanar imaging has led to the increasing adoption of low-dose radiographic techniques in clinical practice, aiming to reduce radiation exposure while preserving sufficient image quality for spinal assessment (Deschênes et al., 2010; Melhem et al., 2016). These systems have been progressively implemented, particularly in the evaluation of spinal deformities such as adolescent idiopathic scoliosis (AIS), where repeated imaging is often required for diagnosis and longitudinal monitoring (Dietrich et al., 2013; Somoskeoy et al., 2012; Illes and Somoskeoy, 2012). Although these advances represent a significant improvement in radiation safety, even low-dose radiographic protocols entail cumulative exposure, especially in younger patients undergoing repeated follow-up examinations (Rose et al., 2023; Garg et al., 2020). Consequently, the development of methods capable of reducing imaging requirements while preserving clinically relevant geometric information remains an important objective.

Within this framework, artificial intelligence (AI), and particularly deep learning techniques, has emerged as a promising strategy for improving both the efficiency and safety of medical imaging workflows. Deep learning models have demonstrated strong performance in tasks such as anatomical landmark detection, automated parameter extraction, and image reconstruction in musculoskeletal radiography (Hosny et al., 2018; Cina and Galbusera, 2024; Bharadwaj et al., 2025; Patel et al., 2025). By automating the extraction of clinically relevant measurements, these approaches can substantially reduce analysis time and operator dependency while maintaining high levels of accuracy. More recently, deep learning methods have been explored for generating or enhancing radiographic images from limited data, thereby potentially enabling further reductions in radiation exposure. With regard to the prediction of spinal parameters from radiographic images, existing studies have primarily focused on estimating parameters within the same imaging plane—either coronal or sagittal (Galbusera et al., 2022; Kim et al., 2023; Lang et al., 2025; Landriel et al., 2024; Harake et al., 2024; Meng et al., 2025; Berlin et al., 2024; Pucciarelli et al., 2025; Zerouali et al., 2023), —rather than across planes, i.e., predicting parameters of one plane from images acquired in another (e.g., sagittal parameters from coronal views, or *vice versa*).

Building on this emerging paradigm, our recent work investigated the use of generative adversarial networks (GANs) to synthesize sagittal radiographic projections from coronal images, with the objective of estimating sagittal alignment parameters from AI-generated views (Bassani et al., 2025). Although the generated sagittal projections appeared visually consistent with real radiographs, the resulting accuracy in measuring sagittal parameters did not reach clinically acceptable thresholds. These findings suggest that image synthesis alone may be insufficient to reliably recover the complex three-dimensional alignment information required for precise spinopelvic measurements.

Motivated by these limitations, the present study explores an alternative deep learning framework based on a multiple-output regression (MOR) architecture designed to directly predict key sagittal spinal and pelvic parameters from coronal radiographs. By bypassing the intermediate step of synthetic image generation and subsequent manual measurement, this approach aims to extract alignment information directly from the available coronal projection data. An adult radiographic dataset was used for model training and validation to evaluate the feasibility and predictive accuracy of this direct estimation strategy. In addition, as a further validation step at the inference stage, the performance of the MOR model was compared with that of the previously developed GAN-based method using a dataset of subjects with AIS, enabling assessment of model adaptability and potential clinical applicability.

## 2 Materials and methods

### 2.1 Datasets

**FIGURE 1:**
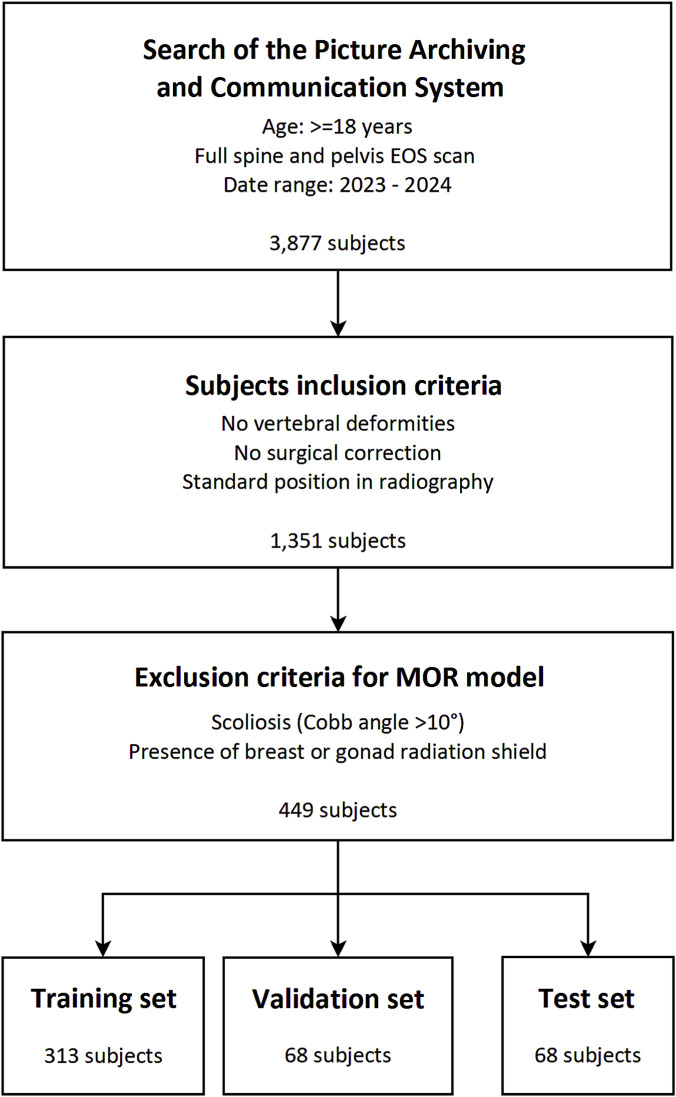
Chart diagram illustrating the workflow for the image selection.

**FIGURE 2:**
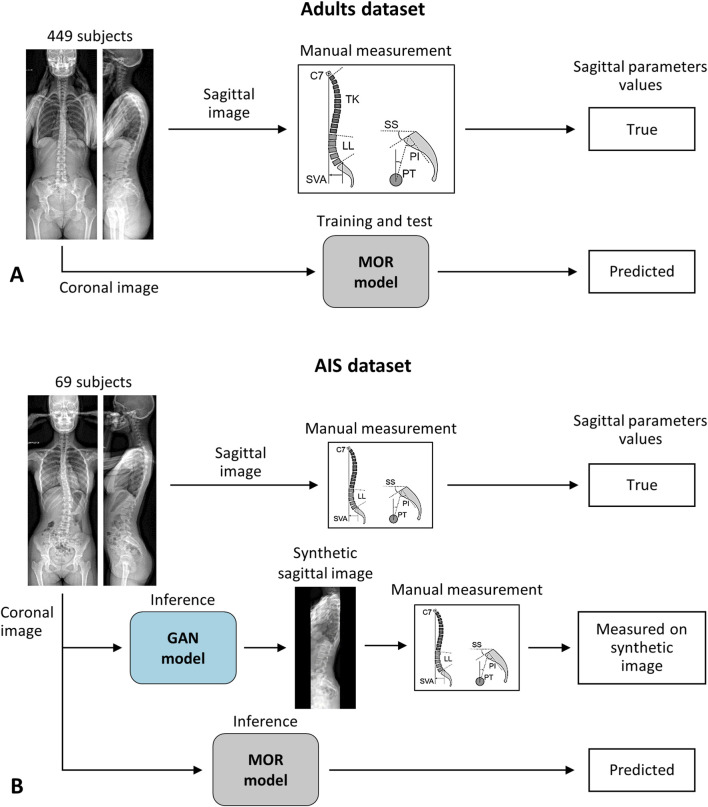
Upper panel **(A)** Schematic representation of the training and testing pipeline for the MOR model using images from the adults dataset, showing the true reference sagittal parameters and the corresponding values predicted by the MOR model. Lower panel **(B)** Schematic representation of the comparison between the MOR- and GAN-based approaches using images from the AIS dataset, showing the true values, the MOR-predicted values, and the values measured on synthetic sagittal images generated by the GAN model.

A retrospective search of the Picture Archiving and Communication System (PACS) of the IRCCS Ospedale Galeazzi-Sant’Ambrogio (Milan, Italy) was performed on anonymized data acquired in the period 2023–2024 (Figure 1). Subjects with the following criteria were included: age larger than 18 years; radiographic examination of the spine and pelvis acquired by the EOS system (EOS Imaging, Paris, France), allowing for the simultaneous acquisition of true size coronal and sagittal images in one-to-one scale avoiding vertical distortion (Figure 2) (Dietrich et al., 2013; Somoskeoy et al., 2012; Illes and Somoskeoy, 2012). Subjects presenting vertebral deformities, those who had undergone operative correction, and those with non-standard positioning during biplanar radiography were excluded. This process yielded a dataset of 1,351 subjects. The largest Cobb angle in the coronal plane, quantifying scoliosis severity, was measured by a panel consisting of two spinal surgeons (RC and MBB, co-authors) and one experienced radiologist (LMS), each with more than 20 years of clinical experience. The dataset was randomly divided into equal subsets among the three evaluators, who independently performed the measurements using the integrated PACS software utility. Images from subjects with scoliosis (Cobb angle >10°) were subsequently excluded, as were images containing breast or gonadal radiation shields, which could not be standardized by the MOR model due to variability in their presence, placement, and size. After these exclusions, the final dataset comprised 449 subjects. The dataset was randomly divided into training (70%, n = 313), validation (15%, n = 68), and test (15%, n = 68) sets. This adults dataset was used to train and evaluate the MOR model (Figure 2A).

In addition, a previously collected dataset of 69 AIS subjects (Cobb angle <45°) was used to compare the MOR and GAN-based approaches (Figure 2B). This cohort had been previously analyzed by our group to evaluate the measurement of sagittal parameters on synthetic sagittal views generated by a GAN model from coronal radiographic images (Bassani et al., 2025). Details of the dataset are reported in Bassani et al. (2025). Briefly, the included subjects were those classified as assessable within a set of 100 randomly selected individuals, based on the visibility of anatomical landmarks required for parameter measurement in the synthetic views. Regarding scoliosis severity, 15 subjects were classified as non-scoliotic (Cobb angle <10°), 42 as having mild scoliosis (Cobb angle 10°–25°), and 12 as having moderate scoliosis (Cobb angle 25°–45°).

### 2.2 Deep learning

The preprocessing pipeline for the coronal radiographic images included the following steps. First, a bounding box encompassing the spine and pelvis—from the superior endplate of C7 to the femoral heads—was manually defined by an experienced biomedical engineer (TB, author) using an in-house application developed in MATLAB (v.R2024b, MathWorks, Natick, MA, United States). The application enabled visualization of the coronal image and manual selection of the region-of-interest boundaries through a standardized and reproducible procedure. Specifically, the centers of the C7 superior endplate and of both femoral heads were identified as anatomical landmarks using a mouse pointer. Adequate inclusion of the femoral heads and proximal femoral shafts was ensured by automatically expanding the bounding box laterally (left and right) and inferiorly by a fixed proportion (0.22) of the horizontal distance between the femoral head centers. This proportional factor was empirically determined on a pilot sample of 25 randomly selected cases. Second, the bounding box coordinates were used to crop the images, which were subsequently inspected to verify the correct inclusion of the aforementioned anatomical structures Third, the cropped images were rescaled to generate two alternative input sizes for the MOR model: 2048 × 1024 pixels and 1024 × 512 pixels. Black padding was added to fill the image width when necessary. Fourth, image contrast was enhanced for both resolutions by saturating the lowest 1% and highest 1% of pixel intensities. Pixel intensities were subsequently normalized to the range 0–1. This preprocessing workflow was used for the adults dataset employed to train and validate the MOR model, as well as for the AIS dataset used to compare MOR-based predictions with those obtained from the previously developed GAN-based model.

For the MOR model, several architectures and hyperparameters were evaluated using the training and validation sets. Three convolutional neural network architectures pretrained on ImageNet were considered: EfficientNetV2-S, ResNet101V2, and DenseNet169. The following hyperparameter ranges were explored: batch size (4, 8, 12, 16), learning rate (10^–3^, 10^–4^, 10^–5^), dropout (0.2, 0.3, 0.4, 0.5), and the proportion of layers unfrozen during fine-tuning (15%, 20%, 25%). The two input image resolutions were evaluated. All combinations of architectures and hyperparameters were assessed. Training was conducted for up to 150 epochs with early stopping based on validation loss (mean squared error), using the Adam optimizer and a patience of 15 epochs. The learning rate was reduced to 10^–7^ when the validation loss plateaued for seven epochs. Cross-validation was not performed due to the limited dataset size. Data augmentation was also not applied in order to preserve the anatomical structure and morphology of the spine in the images. The six target variables were used as raw values (without normalization), and the outputs were equally weighted in the loss function. A constant seed value was applied exclusively for the dataset splitting procedure. All routines for image preprocessing and the implementation of the deep learning models were executed in Python (v.3.10), using the OpenCV library and the TensorFlow Keras (v.2.10) framework (Abadi et al., 2016). Model training was performed using a workstation equipped with an NVIDIA Quadro RTX 5000 GPU (16 GB VRAM).

With regard to the comparison with the GAN-based approach, methodological details are provided in Bassani et al. (2025). Briefly, the generation of a synthetic sagittal image from a coronal view is achieved through two consecutive generative adversarial networks based on the pix2pix framework (Isola et al., 2018; Zhou et al., 2023), both adopting a U-Net architecture. The first GAN takes the coronal image as input to generate a synthetic sagittal image. The resulting image is then used as input to a second network, designed to enhance image contrast and spatial resolution.

It is worth noting that a direct comparison between MOR- and GAN-based approaches was conducted only on the AIS dataset. A similar comparison in the adults dataset was not feasible. Preliminary analyses showed that GAN-generated sagittal images derived from adult coronal radiographs were frequently of insufficient quality, exhibiting limited anatomical detail and inadequate visibility of key vertebral landmarks required for parameter measurement.

### 2.3 Model accuracy and statistical analysis

Six sagittal parameters were independently measured manually by the three evaluators within their respective subsets using the integrated PACS software utility on the original sagittal radiographs to obtain the ‘true’ reference values: thoracic kyphosis (TK), defined as the angle between the line connecting the upper endplate corners of T1 vertebra and the line connecting the lower endplate corners of T12; lumbar lordosis (LL), defined as the angle between the line connecting the upper endplate corners of L1 and the line connecting the lower endplate corners of L5; sacral slope (SS), defined as the angle between the line connecting the corners of the sacral plate and the horizontal reference line; pelvic incidence (PI), defined as the angle between the line perpendicular to the sacral plate at its midpoint and the line connecting this point to the center of the bicoxofemoral axis; pelvic tilt (PT), defined as the angle between the vertical line and the line connecting the sacral midpoint to the center of the bicoxofemoral axis; and sagittal vertical axis (SVA), defined as the horizontal distance between the plumb line dropped from the centroid of the C7 vertebra and the posterior corner of the sacral endplate, where a positive value indicates the line falls anterior to the posterior corner of the sacral endplate (Figure 2). Differences in the median values of age and spinal parameters between females and males were assessed using the Wilcoxon rank-sum test, due to the non-normal distribution of the data, with a significance level of α = 0.05.

The performance of the MOR model in predicting sagittal parameters was evaluated in the test set of adult subjects (Figure 2A). Model performance was assessed through regression analysis and Bland–Altman plots comparing true and predicted values, together with the calculation of mean absolute error (MAE), root mean square error (RMSE), and Lin’s concordance correlation coefficient (CCC) (Akoglu, 2018). The slope and intercept of the regression line were evaluated. In particular, the slope value (i.e., the regression coefficient) was converted into a line angle for geometric interpretation by computing the arctangent of the regression coefficient and multiplying by 180/π to express the result in degrees. Accordingly, a perfect match—indicated by a slope of 1—corresponds to a 45° line angle. This choice was motivated by the aim of facilitating a clearer visual comparison of line slopes in regression plots. It also reflects the intention to make the results more accessible to a broader audience, including readers without a strictly technical or analytical background, such as clinicians and therapists. In the Bland–Altman analysis, a one-sample t-test was used to assess whether the mean difference between true and predicted values was statistically different from zero. Confidence intervals for CCC were estimated using a bootstrap approach. The CCC ranges from 0 to 1, similarly to the Pearson correlation coefficient, but combines precision (Pearson correlation) and accuracy (bias correction) into a single metric. Unlike traditional correlation analysis, CCC is sensitive to systematic bias in magnitude between datasets and therefore provides a more comprehensive measure of agreement, whereas correlation alone only quantifies the strength of a linear relationship.

The same approach was used to compare MOR-based and GAN-based methods using inference on the AIS dataset. The TK parameter was excluded from the analysis because it was not measurable on synthetically generated images due to the frequent absence of visible landmarks for the T1 vertebra, which has been reported as an intrinsic limitation in the reference study describing model development (Bassani et al., 2025). The absolute error between the true parameter values and those predicted by the MOR model was compared with the absolute error between the true values and those measured on the synthetic images generated by the GAN model (Figure 2B). Differences in the median absolute errors between the two conditions (true *minus* predicted vs. true *minus* synthetic) were evaluated using a paired-sample test, given that both conditions share the same underlying true data. Specifically, we applied the Wilcoxon signed-rank test due to the non-normal distribution of the differences. The correlation between the absolute error of each model and the Cobb angle (quantifying scoliosis severity) was assessed using Pearson or Spearman correlation coefficients, with statistical significance evaluated using a t-test or permutation test to determine deviation from zero. A significance level of α = 0.05 was adopted for all analyses. All statistical analyses were performed using MATLAB.

## 3 Results

The adults dataset comprised 449 subjects, with a balanced sex distribution (47% females and 53% males; Table 1). The median (IQR) age was 55 (21) years overall, with a slightly higher median age in females compared with males (61 vs. 50 years). Median values of spinal parameters were generally comparable between sexes, with the exception of PT, which was slightly higher in females than in males (18° vs. 15°).

**TABLE 1:** Age and sagittal alignment parameters in the overall population, and separately in females and males, for the adults and AIS datasets.

Demographic and alignment parameters	All	Females	Males
Adults dataset			
Subjects	449	209 (47%)	240 (53%)
Age (years)	55 (21), 18–87	61 (19), 20–85	50 (24), 18–87*
TK (°)	49 (16), 3–91	50 (17), 12–91	49 (17), 3–84
LL (°)	37 (17), 1–85	38 (15), 1–75	37 (19), 1–85
SS (°)	34 (12), 1–68	33 (11), 5–54	34 (13), 1–68
PI (°)	50 (15), 25–91	50 (15), 25–91	50 (16), 25–86
PT (°)	16 (11), 1–51	18 (12), 1–46	15 (11), 1–51*
SVA (cm)	0.8 (4.3), −7.5–14.5	0.6 (4.5), −6.3–13.3	1 (4.3), −7.5–14.5
AIS dataset			
Subjects	69	24 (35%)	45 (65%)
Age (years)	14 (4), 10–18	13 (4), 10–18	15 (3), 11–18*
LL (°)	44 (15), 18–71	46 (9), 24–65	41 (16), 18–71
SS (°)	36 (11), 22–57	37 (10), 23–57	35 (9), 22–56
PI (°)	41 (13), 29–68	39 (10), 30–68	42 (16), 29–62
PT (°)	6 (9), -19–21	8 (9), -19–17	5 (8), -12–21
SVA (cm)	−1 (3), -6–4	−2 (3), -6–3	−1 (3), -5–4*

Data are reported as number of cases (percentage relative to the total number of subjects within each sex group), median (IQR), and range.

*Indicates a significant difference in median values between females and males: specifically, p < 0.001 and p = 0.002 for age and PT, in adults, and p = 0.028 and p = 0.033 for age and SVA, in AIS. TK, thoracic kyphosis; LL, lumbar lordosis; SS, sacral slope; PI, pelvic incidence; PT, pelvic tilt; SVA, sagittal vertical axis.

In the AIS dataset, which included 69 subjects, the proportion of females was lower than that of males (35% vs. 65%). The median (IQR) age was 14 (4) years overall, and slightly lower in females than in males (13 vs. 15 years). Median spinal parameter values were similar between sexes, with only SVA showing a slightly more negative median value in females compared with males (−2 vs. −1 cm).

### 3.1 MOR model training and test

The optimal model architecture and hyperparameter configuration identified using the training and validation sets from the adults dataset was DenseNet169, with an input image size of 1024 × 512 pixels, a batch size of 8, a learning rate of 1 × 10^−3^, a dropout rate of 0.3, and 25% fine-tuning (Table 2).

**TABLE 2:** Tested architectures and hyperparameters for the MOR model, including all evaluated options and values. The best configuration—defined as the one achieving the lowest loss on the validation set and subsequently used in the study—is indicated with underlining.

Architecture and hyperparameters	Tested options and values
Net architecture (Pre-trained from ImageNet)	efficientnetV2S, resnet101V2, densenet169
Image size (px)	1024x512, 2048x1024
Batch size	4, 8, 12, 16
Learning rate	1e-3, 1e-4, 1e-5
Drop-out rate	0.2, 0.3, 0.4, 0.5
Fine tuning (Percentage of retrained layers)	15%, 20%, 25%

When evaluated on the test set, the best MOR configuration yielded the following median (IQR) absolute errors between true and predicted values: 8 (9)° for TK, 6 (7)° for LL, 5 (6)° for SS, 7 (10)° for PI, 4 (5)° for PT, and 1.6 (2.1) cm for SVA (Table 3). The maximum observed errors ranged from 23° to 40° for the angular parameters, and reached 8.8 cm for SVA. RMSE ranged from 6° to 11° for the angular measures and was 3 cm for SVA. Concordance correlation coefficients (CCC) between true and predicted values were 0.63 (TK), 0.49 (LL), 0.32 (SS), 0.20 (PI), 0.69 (PT), and 0.58 (SVA).

**TABLE 3:** Absolute error, MAE, RMSE, and Lin’s concordance correlation coefficient (CCC) between true and predicted sagittal parameters for the adults dataset.

Performance metrics	TK	LL	SS	PI	PT	SVA
Absolute error (true – predicted)	8 (9), 0°–26°	6 (7), 0°–40°	5 (6), 0°–32°	7 (10), 0°–28°	4 (5), 0°–23°	1.6 (2.1), 0–8.8 cm
MAE/RMSE	9/11°	8/11°	6/8°	8/10°	4/6°	2.2/3 cm
CCC	0.63 (0.44–0.76)	0.49 (0.28–0.7)	0.32 (0.15–0.49)	0.2 (−0.01–0.39)	0.69 (0.54–0.78)	0.58 (0.46–0.67)

Absolute error is given as median (IQR), and range. CCC, is given as coefficient value and confidence interval; TK, thoracic kyphosis; LL, lumbar lordosis; SS, sacral slope; PI, pelvic incidence; PT, pelvic tilt; SVA, sagittal vertical axis.

**FIGURE 3:**
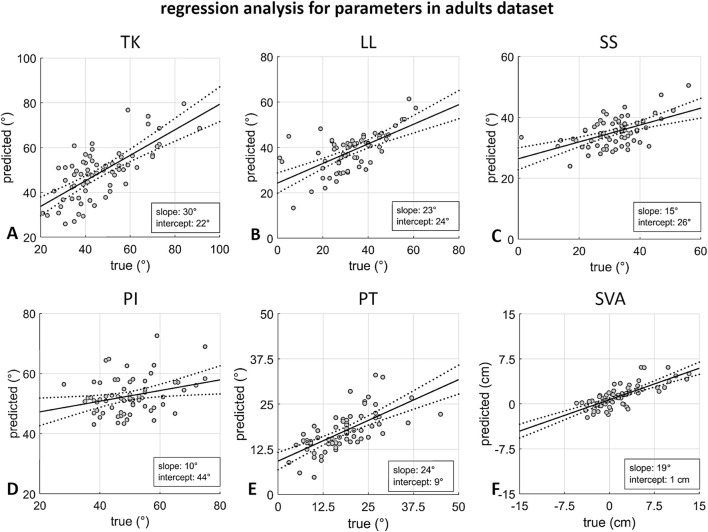
Scatter plots illustrating the relationship between true and MOR-predicted parameter values in the test set from adults dataset, including regression lines, 95% confidence intervals, and the slope and intercept of each regression line. Sagittal parameters shown are: thoracic kyphosis, TK **(A)**; lumbar lordosis, LL **(B)**; sacral slope, SS **(C)**; pelvic incidence, PI **(D)**; pelvic tilt, PT **(E)**; and sagittal vertical axis, SVA **(F)**.

**FIGURE 4:**
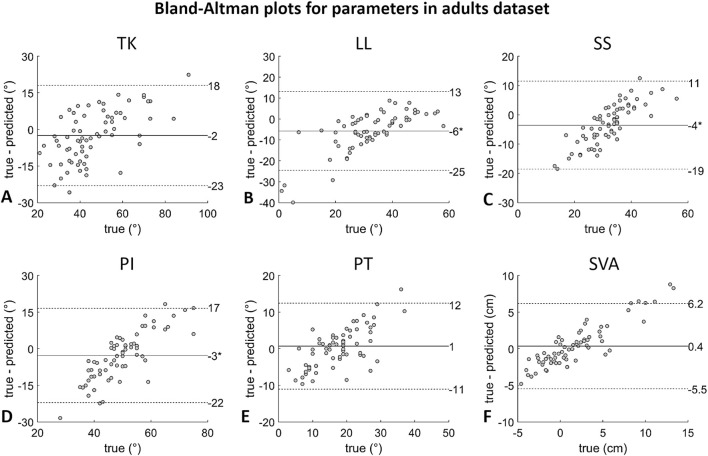
Bland–Altman plots illustrating the relationship between the differences (true minus MOR-predicted values) and the true values in the test set from the adults dataset. Each plot includes the mean difference line (followed by ‘*’ if found significantly different from zero) and the 95% (±1.96 SD) limits of agreement. Sagittal parameters shown are: thoracic kyphosis, TK **(A)**; lumbar lordosis, LL **(B)**; sacral slope, SS **(C)**; pelvic incidence, PI **(D)**; pelvic tilt, PT **(E)**; and sagittal vertical axis, SVA **(F)**.

Regression analysis yielded slope and intercept values of 30° and 22° for TK, 23° and 24° for LL, 15° and 26° for SS, 10° and 44° for PI, 24° and 9° for PT, and 19° and 1 cm for SVA (Figure 3). Bland–Altman analysis indicated that the difference between reference and predicted values increased with deviation from the mean reference value for each spinal parameter (Figure 4). A mean difference significantly different from zero was detected for LL (−6°), SS (−4°), and PI (−3°) based on one-sample t-tests (Figures 4B–D).

### 3.2 Comparison between MOR and GAN-based approaches

Inference on the AIS dataset yielded the following median (IQR) absolute errors between true values and those predicted by the MOR model, and between true values and parameters measured on GAN-generated synthetic images: 7 (8)° and 6 (7)° for LL, 6 (8)° and 7 (10)° for SS, 9 (9)° and 7 (11)° for PI, 6 (8)° and 7 (7)° for PT, and 2 (2) and 1 (1) cm for SVA, the latter showing a significant difference (Table 4). The maximum observed errors ranged from 22° to 35° for MOR-predicted angular values and from 27° to 39° for synthetic-based values. For SVA, maximum errors reached 6 cm and 3.3 cm for predicted and synthetic values, respectively. RMSE ranged from 9° to 13° (true *minus* predicted) and from 10° to 12° (true *minus* synthetic) for the angular parameters, and were 3 cm and 1 cm for SVA, respectively. CCC between true and predicted values, and between true and synthetic values, were respectively: 0.28 and 0.50 (LL), 0.26 and 0.17 (SS), 0.15 and 0.17 (PI), 0.07 and −0.01 (PT), and 0.24 and 0.67 (SVA).

**TABLE 4:** Absolute error, MAE, RMSE, and Lin’s concordance correlation coefficient (CCC) between true and predicted values, and between true values and those measured on synthetic images, for the sagittal parameters in the AIS dataset. The final section reports the correlation coefficient between absolute error and Cobb angle.

Performance metrics	LL	SS	PI	PT	SVA
Absolute error					
True – predicted	7 (8), 0°–25°	6 (8), 0°–22°	9 (9), 0°–31°	6 (8), 0°–35°	2 (2), 0–6 cm
True – synthetic	6 (7), 0°–33°	7 (10), 0°–27°	7 (11), 0°–39°	7 (7), 0°–28°	1 (1), 0–3.3 cm
p-value	n.s	n.s	n.s	n.s	<0.001
MAE/RMSE					
True – predicted	8/10°	7/9°	10/13°	8/10°	2/3 cm
True – synthetic	7/10°	9/11°	9/12°	8/10°	1/1 cm
CCC					
True *and* predicted	0.28 (0.05–0.47)	0.26 (0.04–0.44)	0.15 (0.01–0.31)	0.07 (−0.06–0.21)	0.24 (0.09–0.38)
True *and* synthetic	0.5 (0.31–0.65)	0.17 (−0.06–0.37)	0.17 (−0.05–0.38)	−0.01 (−0.21–0.2)	0.67 (0.52–0.77)
Correlation between absolute error and Cobb angle					
True – predicted	0.03	−0.06	−0.20	0.22	−0.01
True – synthetic	−0.06	−0.06	0.04	0.20	−0.09

Absolute error is given as median (IQR), and range. CCC, is given as coefficient value and confidence interval. N.s., indicates not significant p-value in the comparison between true–predicted and true–synthetic values in the considered parameter. LL, lumbar lordosis; SS, sacral slope; PI, pelvic incidence; PT, pelvic tilt; SVA, sagittal vertical axis.

**FIGURE 5:**
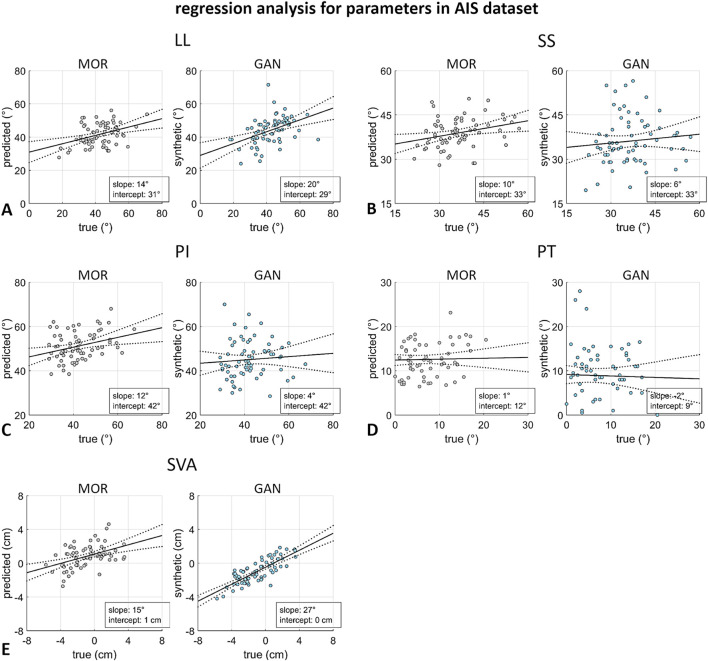
Scatter plots illustrating the relationship between true and MOR-predicted parameter values (light grey) and between true values and those measured on GAN-generated synthetic images (light blue) in the AIS dataset. Each plot includes regression lines, 95% confidence intervals, and the corresponding slope and intercept of the regression line. Sagittal parameters shown are: lumbar lordosis, LL **(A)**; sacral slope, SS **(B)**; pelvic incidence, PI **(C)**; pelvic tilt, PT **(D)**; and sagittal vertical axis, SVA **(E)**.

**FIGURE 6:**
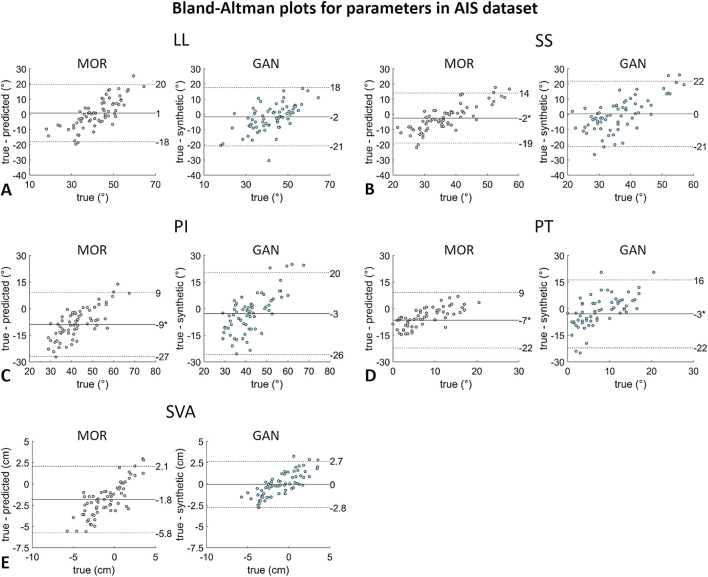
Bland–Altman plots illustrating the relationship between the differences (true *minus* MOR-predicted values, shown in light grey; and true *minus* values measured on GAN-generated synthetic images, shown in light blue) and the true values in the AIS dataset. Each plot includes the mean difference line (followed by ‘*’ if found significantly different from zero) and the 95% limits of agreement (±1.96 SD). Sagittal parameters shown are: lumbar lordosis, LL **(A)**; sacral slope, SS **(B)**; pelvic incidence, PI **(C)**; pelvic tilt, PT **(D)**; and sagittal vertical axis, SVA **(E)**.

Regression analysis yielded slopes ranging from 1° to 15° (true vs. predicted) and from −2° to 27° (true vs. synthetic), while intercepts ranged from 12° to 42° and from 9° to 42°, respectively (Figure 5). For SVA, intercepts were 1 cm and 0 cm, respectively. Bland–Altman analysis confirmed that, for both approaches, differences between true and predicted or synthetic values generally increased with deviation from the mean true values of the spinal parameters (Figure 6). A mean difference significantly different from zero was observed for true vs. predicted values for SS and PI (−2° and −9°, respectively; Figures 6B,C), and for both predicted and synthetic values in the case of PT (−7° and −3°, respectively; Figure 6D).

Regarding the correlation between absolute error and scoliosis severity, the coefficients for the MOR and GAN-based approaches were 0.03 and −0.06 for LL, respectively; −0.06 for both approaches for SS; −0.20 and 0.04 for PI; 0.22 and 0.20 for PT; and −0.01 and −0.09 for SVA (Table 4).

## 4 Discussion

The present study investigated the feasibility of directly estimating sagittal spinopelvic parameters from coronal radiographs by MOR deep learning framework, and compared its performance with a previously developed GAN-based image synthesis approach. Overall, the findings indicate that, despite partial predictive capability for selected parameters, the level of agreement between predicted and true reference values remains insufficient for reliable clinical or biomechanical application. This outcome highlights intrinsic methodological and anatomical limitations that constrain the extraction of sagittal information from coronal projections.

From a quantitative standpoint, the MOR model achieved moderate performance for certain parameters, TK, PT and SVA, as reflected by relatively higher CCC (ranging from 0.58 to 0.69, Table 3). In contrast, other key parameters, including PI and SS, exhibited weak agreement, with low CCC values (0.20 and 0.32, respectively) and substantial dispersion in Bland–Altman analyses (Figure 4). With respect to absolute error, median values for angular parameters ranged from 4° to 8°; however, maximum errors were considerably larger, ranging from 23° (PT) to 40° (LL) (Table 3). These findings indicate that the predictive performance of the model is not uniform across anatomical descriptors and may depend on the degree to which each parameter is indirectly represented within coronal morphology.

Regression analyses revealed both proportional and fixed biases across most parameters (Figure 3). Non-unit slopes (i.e., deviating from 45° when expressed as an angle) indicates proportional bias, whereby prediction errors increase as a function of the magnitude of the true values. This trend was further confirmed by Bland–Altman plots (Figure 4), which demonstrated widening limits of agreement at higher or lower extremes of the measured parameters. In parallel, non-zero intercepts in regression analysis reflect systematic offsets, indicating that the model consistently overestimates or underestimates specific parameters regardless of their magnitude. Such combined biases undermine the trueness and reliability of the predictions, particularly in subjects with pronounced deviations from average alignment.

The observed significant mean differences in Bland–Altman plots for parameters such as LL, SS, and PI further support the presence of systematic errors (Figures 4B–D). Although the magnitude of these biases may appear moderate in absolute terms, their clinical relevance should not be underestimated. In the context of biomechanical modeling, even small angular discrepancies can propagate into significant errors in load distribution, joint mechanics, and muscle force estimation (Barba et al., 2021; Bassani et al., 2019; Bassani et al., 2024; Rauber et al., 2024; Carpenedo et al., 2025). Consequently, the current level of accuracy does not meet the requirements for subject-specific modeling applications.

A direct comparison between MOR- and GAN-based approaches was performed only on the AIS dataset. An analogous comparison on the adults dataset was not feasible, as GAN-generated sagittal images derived from adult coronal radiographs were frequently of insufficient quality. Specifically, these images were characterized by limited anatomical detail and poor visibility of key vertebral landmarks required for parameter measurement, resulting in a very low number of usable cases and precluding a robust comparison. This observation suggests that GAN-based image synthesis may be highly sensitive to dataset characteristics and may exhibit limited adaptability, even within relatively homogeneous adult populations. It is worth noting that unconstrained image-to-image translation models may produce artifacts and blurred anatomical boundaries, potentially limiting GAN performance and affecting the comparison between methods. One possible strategy to address this limitation would be to incorporate explicit anatomical constraints into the GAN training process. Specifically, the model could be modified to simultaneously generate sagittal radiographs and the corresponding vertebral segmentation masks. However, such an approach is beyond the scope of the present work and may be considered in future developments.

Within the AIS cohort, despite fundamental differences in methodology—direct parameter regression versus image synthesis followed by manual measurement—both approaches yielded comparable levels of accuracy for most parameters (Table 4). Neither method demonstrated consistent superiority across all metrics, with median absolute errors for angular parameters generally around 7° for both approaches, and similar maximum errors and RMSE values. However, the GAN-based approach showed superior performance for SVA, with a lower median absolute error (1 cm versus 2 cm for MOR) and a statistically significant difference between the two methods (p < 0.001). This may be attributed to the preservation of visually interpretable spatial cues in the synthetic sagittal projections. This interpretation is further supported by agreement metrics, with slightly higher CCC values observed for the GAN-based approach in the case of LL and SVA (0.50 and 0.67, respectively) compared with the MOR approach (0.28 and 0.24). These differences are also evident in the regression analysis, where a steeper slope was observed for SVA in the GAN-based approach compared with MOR (27° versus 15°, Figure 5E), and indicating improved proportional agreement. Consistently, Bland–Altman analysis showed a narrower distribution of differences for the GAN-based method in the case of SVA (Figure 6E), suggesting reduced variability and improved agreement relative to the MOR approach for this specific parameter.

The external inference analysis conducted on the AIS dataset further reinforces this conclusion. Both MOR and GAN-based methods exhibited reduced performance in this cohort compared with the adult dataset, as evidenced by lower CCC values and higher variability. This trend is also reflected in the regression analysis for the MOR approach, where the slopes are markedly lower in the AIS dataset, ranging from 1° to 15° (Figure 5). This degradation can be attributed to several factors, including differences in anatomy, growth-related variability, and the presence of deformities that alter the relationship between coronal and sagittal alignment. The MOR model was trained on adult subjects after excluding those with scoliosis greater than 10°, thereby introducing an age- and pathology-related domain shift when applied to adolescents with scoliosis. The limited transferability of the models across populations underscores the challenges associated with learning generalized mappings between orthogonal imaging planes. Moreover, it is noteworthy that, although AIS is known to be more prevalent and generally more severe in females than in males, the evaluated dataset showed a predominance of male subjects. This imbalance is attributable to the case selection process in the original dataset, in which female radiographs containing breast radiation shields were excluded due to the inability of the generative model to standardize their presence, position, and size (Bassani et al., 2025). Additionally, the selection of assessable GAN-generated images yielded more consistent image quality for male subjects. Regarding the impact of deformity, both methods demonstrated a generally weak correlation between absolute error and scoliosis severity, with correlation coefficients below 0.20 and 0.22 in absolute value for the MOR and GAN approaches, respectively (Table 4). These findings confirm, also for the MOR model, the observation previously reported for the GAN model (Bassani et al., 2025), namely, that the difficulty in generating clinically relevant sagittal images or directly predicting sagittal parameters is not strongly influenced by the degree of spinal deformity in the coronal plane.

Overall, these findings suggest that the primary limitation is not related to the specific deep learning architecture employed, but rather to the underlying information content of the input data. A key methodological distinction between the MOR and GAN-based approaches lies in the way sagittal information is inferred from coronal data. The MOR framework, implemented using a DenseNet169 architecture (Table 2), performs a direct mapping between image features and numerical outputs, effectively learning a global, implicit representation of the relationship between coronal appearance and sagittal alignment parameters. In contrast, the GAN-based approach relies on a U-Net generator to produce a synthetic sagittal image from the coronal input, followed by explicit parameter extraction through manual landmark identification (Bassani et al., 2025). Despite these differences, both approaches ultimately depend on the same underlying information source—the coronal projection—and therefore share a common limitation in terms of available anatomical content. The MOR model compresses this information into abstract feature representations optimized for regression, whereas the GAN attempts to reconstruct a visually plausible intermediate representation before measurement. The comparable performance observed between the two methods suggests that the bottleneck is not the learning paradigm (regression versus image synthesis), but rather the insufficiency of coronal features to encode subject-specific sagittal geometry with high fidelity. While certain indirect correlations between coronal and sagittal features may exist, they appear insufficient to support robust and generalizable predictions. This interpretation is consistent with the observation that prediction errors increase for values farther from the population mean, where individual anatomical variability becomes more pronounced and less inferable from coronal features alone.

Several methodological aspects of the study should also be considered when interpreting the results. Regarding the true measured parameters, no consensus-based assessment or evaluation of inter-rater reproducibility among the three observers was performed. This choice was justified by the extensive clinical experience of all observers and by the fact that the spinal parameters measured are simple and routinely obtained, with reliability coefficients widely reported in the literature as generally high and reproducible. Regarding model training, the absence of data augmentation, although justified by the need to preserve anatomical fidelity, may have limited the robustness of the trained models. Similarly, the relatively small dataset size constrained the exploration of more complex architectures and prevented the use of cross-validation strategies. Nonetheless, these limitations are unlikely to fully account for the observed performance gaps, which appear primarily driven by intrinsic data constraints rather than model capacity. Regarding the model architecture, we opted to use pre-trained CNN architectures rather than more advanced approaches, such as Vision Transformers or state-space models (e.g., Mamba-based architectures). CNN-based architectures have consistently demonstrated strong performance in medical imaging tasks, including radiographic analysis and regression-based prediction of anatomical and biomechanical parameters, particularly in scenarios characterized by limited data availability. In contrast, transformer-based models generally require extensive pre-training and substantially larger datasets to fully leverage their representational capacity. Similarly, recently proposed Mamba-based architectures, although promising, remain comparatively less validated in medical imaging applications and often require extensive hyperparameter optimization and large-scale training conditions. The available computational infrastructure consisted of a workstation equipped with a 16 GB GPU, imposing practical constraints on the training of large transformer-based or hybrid architectures, especially when processing high-resolution radiographic images. Under these hardware and dataset limitations, the use of pre-trained CNN models represented a more computationally efficient and methodologically appropriate solution for MOR training.

From a clinical and biomechanical perspective, the results indicate that current deep learning approaches cannot replace biplanar imaging for the accurate assessment of sagittal alignment. While low-dose imaging systems already mitigate radiation exposure, further reductions through the elimination of one projection are not currently feasible without compromising measurement reliability. Future research may explore alternative strategies, such as the integration of additional data modalities (e.g., 3D surface topography or prior anatomical models), or the development of hybrid physics-informed learning frameworks that incorporate biomechanical constraints.

In conclusion, both MOR-based direct regression and GAN-based image synthesis approaches demonstrate limited capability in reconstructing sagittal alignment from coronal radiographs. The persistence of systematic and proportional biases, coupled with poor agreement for several key parameters, suggests that sagittal information cannot be reliably inferred from coronal projections alone. These findings emphasize the need to reconsider the assumptions underlying cross-plane prediction and to explore more comprehensive approaches for reducing imaging requirements without sacrificing accuracy.

## Data Availability

The data analyzed in this study is subject to the following licenses/restrictions: The datasets used and/or analysed during the current study are available from the corresponding author on reasonable request. Requests to access these datasets should be directed to Tito Bassani, tito.bassani@grupposandonato.it.
